# Discrepancy in the Usage of GFAP as a Marker of Satellite Glial Cell Reactivity

**DOI:** 10.3390/biomedicines9081022

**Published:** 2021-08-16

**Authors:** Kjeld Morten Mohr, Lone Tjener Pallesen, Mette Richner, Christian Bjerggaard Vaegter

**Affiliations:** Danish Research Institute of Translational Neuroscience (DANDRITE), Nordic-EMBL Partnership for Molecular Medicine, Department of Biomedicine, Aarhus University, 8000 Aarhus, Denmark; kjeldmorh1@gmail.com (K.M.M.); ltp@biomed.au.dk (L.T.P.); mette.richner@biomed.au.dk (M.R.)

**Keywords:** satellite glial cells, GFAP, dorsal root ganglion, nerve injury, inflammation

## Abstract

Satellite glial cells (SGCs) surrounding the neuronal somas in peripheral sensory ganglia are sensitive to neuronal stressors, which induce their reactive state. It is believed that such induced gliosis affects the signaling properties of the primary sensory neurons and is an important component of the neuropathic phenotype leading to pain and other sensory disturbances. Efforts to understand and manipulate such gliosis relies on reliable markers to confirm induced SGC reactivity and ultimately the efficacy of targeted intervention. Glial fibrillary acidic protein (GFAP) is currently the only widely used marker for such analyses. However, we have previously described the lack of SGC upregulation of GFAP in a mouse model of sciatic nerve injury, suggesting that GFAP may not be a universally suitable marker of SGC gliosis across species and experimental models. To further explore this, we here investigate the regulation of GFAP in two different experimental models in both rats and mice. We found that whereas GFAP was upregulated in both rodent species in the applied inflammation model, only the rat demonstrated increased GFAP in SGCs following sciatic nerve injury; we did not observe any such GFAP upregulation in the mouse model at either protein or mRNA levels. Our results demonstrate an important discrepancy between species and experimental models that prevents the usage of GFAP as a universal marker for SGC reactivity.

## 1. Introduction

Satellite glial cells (SGCs) are supportive cells intimately enveloping the somata of peripheral sensory and autonomic neurons and structurally organized with several SGCs surrounding a single neuronal soma, forming a functional SGC–neuron unit [1]. SGCs are thus ideally positioned to partake in neuronal homeostasis and signaling. The close monitoring of neuronal homeostasis by SGCs is revealed by their adaptive changes following neuronal injury or stress. Accordingly, a wide range of neuronal stressors linked to the development of acute or chronic pain conditions are accompanied by molecular and cellular changes of SGCs in the affected SGC–neuron units [2]. Such SGC responses have been confirmed in rodent models of systemic and local inflammation [3,4,5], diabetes [6,7,8,9], chemotherapy [10,11], and viral infection [12] as well as after variations of traumatic injury to peripheral nerves ([13,14,15] and are reviewed in [1,2]). Interestingly, pharmacological studies in vitro and in vivo have demonstrated how SGCs constitute a potential new target for the treatment of peripheral neuropathic pain [2,16]. The success of this approach is challenged by the rather limited knowledge of several fundamental aspects of SGC function and details of their response to neuronal stress. A significant barrier is the structure and position of the SGCs forming a relatively thin envelope around the neuronal soma, making the study of SGCs very challenging in tissues with standard microscopy techniques. Studying the SGC reactive response in vitro poses an even greater challenge due to their tendency to rapidly dedifferentiate when cultured [17,18]. Therefore, it is paramount that suitable molecular markers are available to identify SGCs in their reactive state in sectioned tissue obtained from rodent models. Glial fibrillary acidic protein (GFAP) is one such widely used marker in the literature [6,13,19,20,21]. However, we have previously described how we were unable to confirm GFAP upregulation in a mouse model of sciatic nerve ligation [14], suggesting that GFAP upregulation in SGCs is not necessarily observed following nerve injury and thus questions the current usage of GFAP as a universal marker of SGC reactivity. In this work we investigated this hypothesis further and evaluated the GFAP response in two distinct models, the sciatic nerve ligation model representing chronic and defined nerve injury [14,22] and the lipopolysaccharide (LPS)-induced transient systemic inflammation model [3,4], in both mice and rats.

## 2. Materials and Methods

### 2.1. Animals

Eight- to twelve-week-old female C57BL/6J mice and female Sprague Dawley rats, both from Janvier Labs, were used in the study. Animals were housed under standard conditions with a 12 h light/dark cycle and free access to water and standard chow. All animal experiments were performed in full compliance with Danish regulations and all experiments were approved by the Danish Animal Experiments Inspectorate under the Ministry of Environment and Food (permission numbers 2017-15-0201-01192 and 2016-15-0201-01085).

### 2.2. Surgery and Systemic Inflammation Models

Full sciatic nerve ligation was performed under deep isoflurane anesthesia (IsoFlo vet, Zoetis, Kalamazoo, MI, USA). The left sciatic nerve was exposed after skin incision and blunt dissection of the overlying muscles. A tight ligation around the sciatic nerve was made using nonabsorbable 6.0 vicryl suture and the skin was closed with surgical tissue adhesive (Henkel Indermil Tissue adhesive, Farla Medical Ltd., London, UK). For local analgesia, lidocaine (Teva, Haarlem, The Nederlands, 583363) was applied on the wound. Buprenorphine (Temgesic, Schering-Plough, 519752) and ampicillin (Pentrexyl, Applichem, Darmstadt, Germany, A0839,0100) were mixed and diluted in isotonic saline and injected subcutaneously following surgery (rats: Temgesic 0.05 mg/kg and Pentrexyl 50 mg/kg; mice: Temgesic 0.10 mg/kg and Pentrexyl 100 mg/kg). Lumbar dorsal root ganglia (DRGs) were dissected from naïve animals and from the ipsilateral side on days 3 and 14 after surgery, three animals per condition [23].

LPS (Sigma-Aldrich, St. Louis, MO, USA, L2880) was administered by a single intraperitoneal injection. Doses were 2 mg/kg for rats and 2.5 mg/kg for mice dissolved in isotonic saline (injection volumes of 0.50 and 0.25 mL, respectively). Lumbar DRGs were dissected 3 days after the injection, two animals per condition.

Surgery and LPS injections were performed in four independent experiments (*n* = 4) with two animals per condition for each experiment.

### 2.3. Immunohistochemistry

Animals were deeply anesthetized with isoflurane and transcardially perfused with PBS (15 mL/mouse, 50 mL/rat). The DRGs of L3 + L4 in mice and L4 + L5 in rats were identified and dissected by counting from costae [23]. These DRGs were chosen, as they contribute mainly to the sciatic nerve [24]. DRGs were post-fixed in 4% PFA (Sigma-Aldrich, 252549 diluted in PBS) for 2 h at room temperature followed by cryoprotection in 30% (*w/v*) sucrose in PBS overnight at 4 °C. The tissue was embedded in Tissue-Tek (Sakura, 4583) and snap-frozen on dry ice. The DRGs were cut into 10 μm sections using a Leica CM1900 cryostat and stored at −80 °C. The sections were thawed, washed in PBST (PBS with 0.15% (*v/v*) Triton X-100) and blocked with 4% (*v/v*) donkey serum (S30-M, Sigma-Aldrich) in PBST for 1 h at room temperature. The slides were washed in PBST and incubated overnight at 4 °C with primary antibodies diluted in PBST (Table 1). The next day, the slides were washed with PBST followed by incubation for 4 h at room temperature with secondary antibodies diluted in PBST (Table 1). Finally, the sections were washed in PBST with Hoechst nuclear marker (Sigma-Aldrich, 33258) diluted 1:30,000 and mounted with fluorescence mounting medium (Dako, Glostrup, Denmark, S3023). The stained tissues were analyzed using a Zeiss LSM780 confocal microscope, and all images were obtained using identical microscope settings. Images were further processed with the Zen software (Black Edition, and Blue 3.0, Zeiss) to adjust the GFAP intensity threshold using a vague neuronal staining as the background reference signal, as this was assumed to be constant due to lack of neuronal GFAP expression. Alternatively, equal settings across samples, thus disregarding minor sample-to-sample variation, gave similar results. Negative controls were prepared by omitting the primary antibodies (data not shown).

## 3. Results

**Figure 1:**
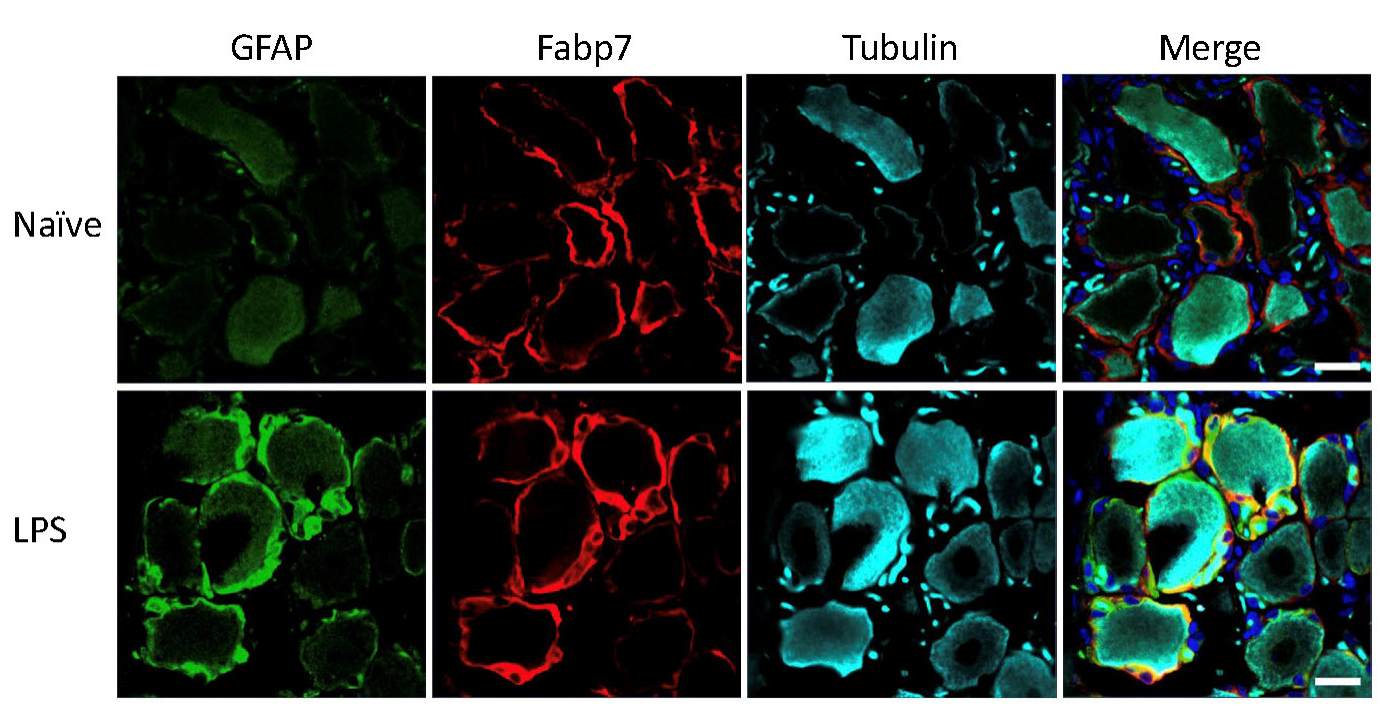
Rat lumbar DRG following LPS treatment. GFAP (green), SGC marker Fabp7 (red), neuronal b-tubulin (magenta), and Hoechst nuclear marker (dark blue, merge only). Scale bar = 20 μm.

**Figure 2:**
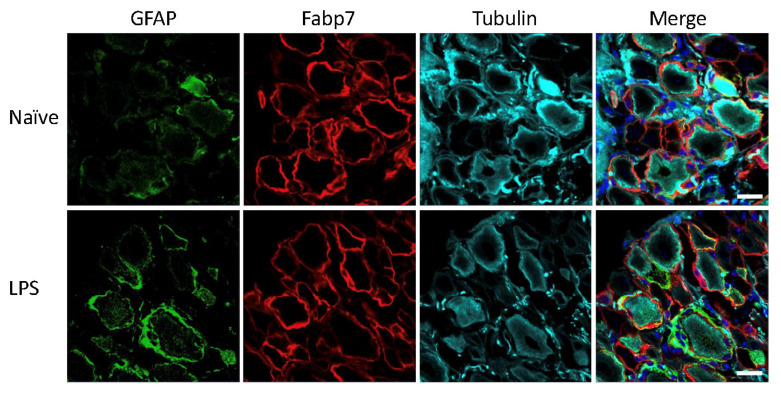
Mouse lumbar DRG following LPS treatment. GFAP (green), SGC marker Fabp7 (red), neuronal b-tubulin (magenta), and Hoechst nuclear marker (dark blue, merge only). Scale bar = 20 μm.

Mice and rats received an intraperitoneal injection of LPS for induction of systemic inflammatory pain, previously described to induce activation of SGCs in mouse sensory ganglia with upregulation of GFAP immunoreactivity, increased sensitivity to ATP, as well as increased SGC-GC coupling via gap junctions [3,4]. To evaluate the effect of the LPS model on GFAP expression in mice and rats, the polyclonal anti-GFAP antibody from Dako was used, which is widely used in other studies to visualize GFAP expression and regulation by immunohistochemistry across species and experimental models [3,4,6,10,14,25,26,27,28,29,30,31,32,33,34,35,36,37,38,39,40,41,42,43,44,45,46,47,48]. The analysis of lumbar DRG sections demonstrated minimal GFAP immunoreactivity in the naïve animals, particularly in the rat (Figure 1) whereas we observed some sporadic signal in mouse lumbar DRGs which seemed to be mainly unspecific neuronal binding (Figure 2). We observed a clear increase in GFAP immunoreactivity in both rat (Figure 1) and mouse (Figure 2) SGCs 3 days after LPS administration, as confirmed by overlay with the SGC marker Fabp7 [15]. GFAP thus appears to be a qualified marker for SGC reactivity following inflammation, as demonstrated by others in both mice [3,4,36] and rats [5,35,49].

**Figure 3:**
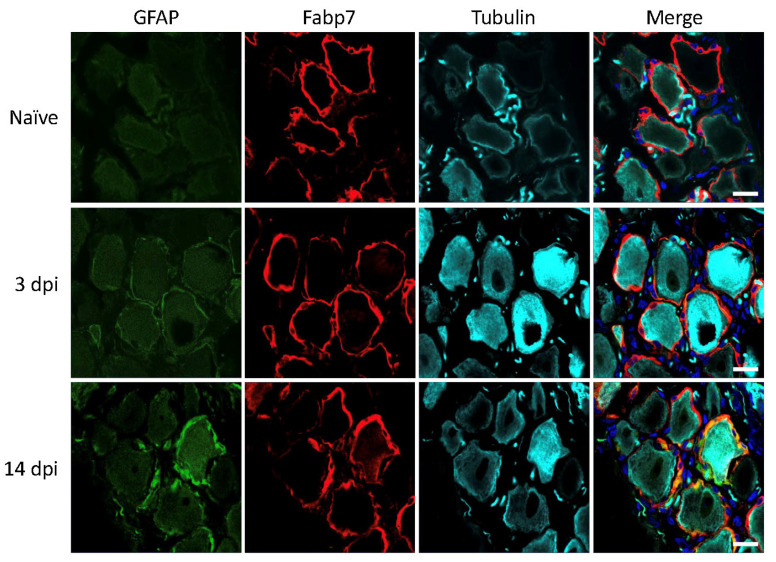
Rat lumbar DRG 3- and 14-days post injury (dpi). GFAP (green), SGC marker Fabp7 (red), neuronal b-tubulin (magenta), and Hoechst nuclear marker (dark blue, merge only). Scale bar = 20 μm.

Next, we tested how traumatic injury to the sciatic nerve affected GFAP expression in the contributing lumbar DRGs [24]. In rats, a clear increase in GFAP immunoreactivity at 3 and 14 days after injury was observed (Figure 3). This is consistent with abundant existing literature on the use of increased GFAP immunoreactivity as a marker for SGC reactivity following traumatic nerve injury in rats (see discussion for details), qualifying the usage of GFAP in this setting.

**Figure 4:**
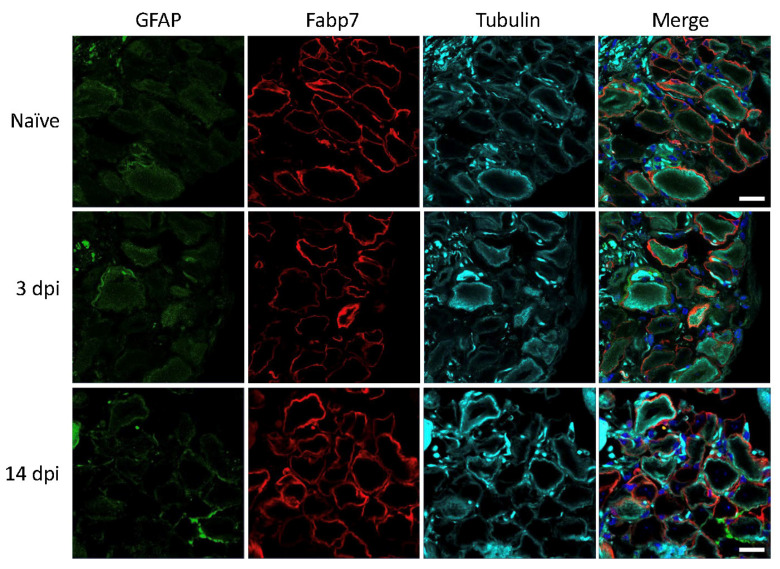
Mouse lumbar DRG 3- and 14-days post injury (dpi). GFAP (green), SGC marker Fabp7 (red), neuronal β-tubulin (magenta), and Hoechst nuclear marker (dark blue, merge only). Scale bar = 20 μm.

In contrast to the results in rats, we did not be observe any increase in GFAP immunoreactivity in SGCs of mouse lumbar DRGs at 3 and 14 days after nerve injury (Figure 4). This is in accordance with results in our previous study under similar conditions [14]. Based on the present LPS studies in mice as well as existing literature, there is very little doubt that the polyclonal anti-GFAP antibody from DAKO is specific and sufficiently sensitive to detect cellular GFAP expression.

Supporting a lack of GFAP upregulation in SGCs is our recent study on the SGC transcriptional response in DRG from mice subjected to sciatic nerve injury. In this study, glutamine-synthetase-positive SGCs from L3-L5 DRGs were FACS isolated for downstream mRNA sequencing by NGS. Despite a comfortable sequencing depth, we did not detect GFAP transcripts above cutoff in SGCs isolated from either naïve mice or 3 and 14 days following injury [14]. We also obtained single cell RNA sequencing data from mouse L3–L4 DRGs in naïve mice as well as 7 and 14 days following partial sciatic nerve ligation (unpublished results), confirming GFAP mRNA expression in only a few percent of SGCs from naïve mice and no significant upregulation of GFAP expression in SGCs 7- and 14-days following nerve ligation (single cell data available at the Gene Expression Omnibus: GSE174430). This is in contrast to previous studies in rat nerve injury models detecting 3–7-fold upregulation of GFAP gene expression in the relevant DRGs [50,51,52].

The data presented here support the notion that mouse SGCs do not express significant levels of GFAP under naïve conditions and that nerve injury (in contrast to inflammation) does not reliably result in an increased GFAP expression level in these cells.

## 4. Discussion

Here we demonstrate, by comparative analysis of two different experimental models in two different rodent species, that SGC reactivity and increased GFAP expression are not causally linked. While both the experimental model in the rat and the inflammation model in the mouse resulted in increased GFAP immunoreactivity of SGCs in the DRGs, the mouse sciatic nerve injury model did not induce increased GFAP immunoreactivity or GFAP gene expression in the corresponding SGCs of DRGs.

GFAP has become a commonly used marker protein to characterize a reactive state of SGCs across species and experimental paradigms. We are unaware of the origin of this approach, however, speculate that it arises from the common functional comparison between CNS astrocytes and PNS SGCs. Despite their rather different developmental origin as well as structural features, both cell types are considered important supportive cells, supporting neuronal homeostasis and activity [21]. An overwhelming amount of evidence exists for the upregulation of GFAP in astrocytes in numerous CNS disease models, and the use of biofluid GFAP levels as a potential biomarker for such diseases has been suggested [53,54,55]. Indeed, GFAP is an intriguing protein. It is an intermediate filament expressed in most glial cells, involved in their cytoskeletal structure as well as their support of neighboring neurons. GFAP is encoded by a single gene but at least ten isoforms with distinct expression patterns have so far been identified. As GFAP (together with vimentin) appears to be a key component in the assembly of intermediate filaments inside astrocytic processes, GFAP seems to play a central role in normal astrocytic function, and the observed GFAP upregulation is believed to be a functionally important part of astrogliosis [53]. Thus, it is very intriguing that several studies on independent GFAP knockout mouse lines have described how these mice seem to be normal in terms of development, growth, fertility, and lifespan. There also seemed to be no significant changes in terms of brain architecture including numbers of neurons and astrocytes, no apparent impact on the function of the blood–brain barrier (as judged by electron microscopy analyses), and no compensatory increase in any other intermediate filaments was observed upon GFAP gene ablation [56]. Even the astrocytic response to CNS injury was largely unaffected in the absence of GFAP [57].

The role of GFAP in the PNS is even less clear compared to the CNS. Studies have described how GFAP is expressed relatively late in Schwann cell development and is downregulated in myelinating Schwann cells while remaining in non-myelinating (Remak) and repair Schwann cells [58,59]. As for the CNS, studies on GFAP knockout mice found that the PNS developed normally and displayed similar (although slightly delayed) nerve regeneration properties compared to wild-type controls [60,61].

Despite this apparent gap in the understanding of the precise mechanistic role of GFAP in the glial reactive response, it appears to serve well as a molecular marker of such glial activation. This property has been utilized to demonstrate SGC activation in rats in various traumatic nerve injury models [13,19,25,26,27,28,29,30,45,47,48,62,63,64,65,66,67,68,69,70,71,72,73,74,75,76,77,78,79,80,81] as well as models of type 1 diabetes [82], ischemia [83], facial cancer [84], chemotherapy administration [33,34], and peripheral inflammation [5,35,49]. Though far from exhaustive, this list of studies demonstrates a strong correlation between increased GFAP immunoreactivity across rat experimental models and induction of SGC reactivity.

For mice, the picture is slightly different. Unfortunately, a vast majority of published papers appear not to distinguish between findings in rats and mice nor experimental models when referring to increased GFAP immunoreactivity as a marker of SGC reactivity. This leads to the conclusion that the generalized link between GFAP and gliosis that we demonstrate here might not always be the case. The literature on mouse models shows increased GFAP expression in models of type 1 diabetes [6,39], chemotherapy [10], and inflammation [3,4,37,38]. However, in stark contrast to rat models we were able to locate very few papers utilizing mouse models of nerve injury with simultaneous analysis of GFAP regulation in the DRGs. Studies by Ohtiro et al. [85] and Lim et al. [86] both show GFAP upregulation in an SGC-like pattern following sciatic/spinal nerve injury. In contrast to the abovementioned mouse models on diabetes, chemotherapy, and inflammation performed in BALB/c or C57BL/6 strains, these two studies apparently used other mouse strains but the provided information is vague. A further study by Zhang et al. identified injury-induced GFAP immunoreactivity in a SCG-like pattern following spared nerve injury. Further staining identified a population of these cells as BrdU+, arguing that they constitute proliferating SGCs [36]. We have previously demonstrated that SGCs do not appear to proliferate following nerve injury. Rather, nerve injury triggers the approximation of (proliferating) macrophages to an SGC-like position surrounding the neuronal somas [14]. The omission of appropriate SGC markers (e.g., glutamine synthetase (GS) or Fabp7) to positively identify perineuronal cells as SGCs may unfortunately give rise to the interpretation of proliferating (BrdU+) cells as SGCs in response to nerve injury (see [14] for details).

Support of absent GFAP regulation in mouse traumatic nerve injury models is evident from other studies. A recent study finds no regulation of GFAP immunoreactivity in SGCs of DRGs following sciatic nerve crush [87], in accordance with observations from our lab in a mouse sciatic ligation model [14]. In the latter study we were further unable to detect any expression of GFAP mRNA in naïve or injured mice (https://rna-seq-browser.herokuapp.com, accessed on 13 August 2021), and in the present manuscript we further substantiate this finding by single-cell analysis of DRGs in the spared nerve injury model. Further, recent transcriptional analyses of the mouse DRG injury response at a single-cell level support our data: a study on the SGC transcriptional response to sciatic nerve crush did not support significant upregulation of GFAP expression using single-cell analysis (https://mouse-drg-injury.cells.ucsc.edu, accessed on 13 August 2021) [15], and similar results can be extracted from single nuclei transcriptional analyses of DRGs obtained from five different models of PNS injury or induced pain (https://painseq.shinyapps.io/publish, accessed on 13 August 2021) [88]. While we might have missed other mouse studies, the data presented here does indeed demonstrate that the universal usage of GFAP upregulation as a molecular marker of SGC reactivity across species and experimental models needs to be reconsidered.

The vast majority of studies described here find little or no GFAP expression in rat or mouse SGCs under naïve conditions. The same appears to be the case for the guinea pig [43], whereas basal expression levels were detected in the dog and monkey [40,89,90]. The purpose of animal models is often to deduce mechanistic insight of relevance to human diseases, but due to the general unavailability of human DRGs we still know relatively little about this tissue [91]. For ethical reasons human nervous tissue can mainly be derived from deceased individuals, or alternatively from surgery. Such studies do demonstrate that SGCs from human DRGs express some level of GFAP [92,93] although information of basal versus induced levels can hardly be derived. Any further comparison of similarities and discrepancies between rodent and human DRGs in general and SGCs in particular therefore remains to be studied.

While such species/model discrepancies can be important for studies pursuing information on SGC reactivity and attempts to therapeutically reduce such an induced phenotype, they may also impact on our understanding of other aspects of SGC biology by the utilization of genetic models. Xiang and colleagues investigated rat SGC transduction by AAV by driving gene expression under the GFAP promotor for glial selectivity. Reporter expression in an SGC-like pattern did indeed confirm successful transduction and is suggestive of some level of baseline GFAP promotor activity in rats, or perhaps reflects a virus-induced reactive state [94]. Based on the transcriptional data discussed here, such a GFAP-based strategy may be directly applicable to some mouse models with confirmed GFAP expression but not to others. For example, a few researchers have studied the NF-κB pathway in SGCs by overexpression of a dominant negative form of the inhibitor of kappa B (IκBα) under the GFAP promotor [95,96]. Fortunately, these studies exploited the chronic constriction model with significant elements of inflammation [97] which as discussed above may induce GFAP expression in mouse SGCs. The choice of e.g., the sciatic ligation model with no underlying induced GFAP expression could likely have interfered with correct interpretations of the NF-κB pathway in SGCs.

A general drawback in numerous studies is the lack of appropriate SGC markers, with the identification of SGCs as cells in very close proximity to the neuronal soma. While SGCs are indeed found at this position [98], the reverse may not always be true, i.e., not all cells in close proximity to the neuronal soma are SGCs. This is evident when observing the migration of macrophages following nerve injury, moving to a position very similar to that of SGCs where they apparently intermingle and make it impossible to differentiate between these two cell types by light microscopy without the use of molecular markers [14]. We strongly urge the use of validated SGC markers such as GS [99] or Fabp7 [15] for future studies. Another common source of confusion is the lack of appropriate identifiers for the antibodies used. The various GFAP studies discussed here have used anti-GFAP antibodies from >11 sources. Sensitivity and specificity [100] are of paramount importance in these studies, but too often the catalogue numbers are omitted which prevents other scientists evaluating the study design by gaining access to prior studies using these antibodies (e.g., provided via www.citeab.com, accessed on 13 August 2021). Some companies provide several different anti-GFAP antibodies, and some antibodies provided across companies/brands may even be identical but distributed under different names. Considering that the main conclusions of the majority of the discussed papers are derived directly from staining patterns, it is crucial that such reagent details are transparent by providing the unique Research Resource Identifier (RRID) provided at www.rrids.org, accessed on 13 August 2021.

In this study we investigated whether an increase in GFAP immunoreactivity is a suitable marker for the detection of SGC reactivity across two different experimental models in both rat and mouse. We found that whereas rat SGCs seemed to indeed increase GFAP expression upon neuronal stress, the same is not true in the mouse where we did not observe any increased GFAP expression following sciatic nerve injury. A closer examination of published studies appears to support this conclusion, suggesting that GFAP may indeed be a suitable SGC reactivity marker for most published experimental models but that caution should be taken when examining, for instance, the mouse injury models. This study is limited to the Sprague Dawley rat strain and the C57BL/6 mouse strain, two commonly used rodent models in experimental research. Further, we have only investigated SGCs in lumbar DRGs. The regulation of GFAP in other rodent strains as well as non-rodent species, not to mention SGCs in sympathetic and trigeminal ganglia, remains to be investigated.

## Figures and Tables

**Table 1:** Antibodies.

Antibodies	Supplier, Cat.	RRID	Dilution
Rabbit anti-GFAP	Dako/Agilent, Z0334	AB_10013382	1:500
Goat anti-Fabp7	R&D Systems, AF3166	AB_2100475	1:350
Mouse anti-neuronal Class III β-tubulin	BioLegend, MMS-435P	AB_2313773	1:1000
Donkey anti-rabbit IgG, Alexa Fluor 488	Invitrogen, A21206	AB_2535792	1:300
Donkey anti-goat IgG, Alexa Fluor 568	Invitrogen, A11057	AB_142581	1:300
Donkey anti-mouse IgG, Alexa Fluor 647	Invitrogen, A31571	AB_162542	1:300

## Data Availability

The data generated for this study are available on request to the corresponding author.
